# The complete sequence of the chloroplast genome of *Chrysanthemum rupestre*, a diploid disciform capitula species of *Chrysanthemum*

**DOI:** 10.1080/23802359.2022.2057252

**Published:** 2022-04-01

**Authors:** Yu Masuda, Michiharu Nakano, Makoto Kusaba

**Affiliations:** a Department of Plant Science, School of Agriculture, Tokai University, Kumamoto, Japan; b Faculty of Agriculture and Marine Sciences, Kochi University, Nankoku, Japan; c Graduate School of Integrated Sciences for Life, Hiroshima University, Higashi-Hiroshima, Japan

**Keywords:** *Chrysanthemum rupestre*, *Ajania*, chloroplast genome

## Abstract

In this study, we analyzed the complete sequence of the chloroplast genome of *Chrysanthemum rupestre* Matsum. et Koidz., 1910, a diploid disciform capitula species of *Chrysanthemum* endemic to Japan, formerly classified as *Ajania rupestris* (Matsum. & Koidz.) Muldashev, Bot. Zhurn. (Moscow & Leningrad), 1983. The chloroplast genome of *C. rupestre* has a typical conserved quadripartite structure of 151,061 bp in length, comprising a large single-copy region (82,846 bp), a small single-copy region (18,301 bp), and a pair of inverted repeat regions (each 24,957 bp). Phylogenetic analysis indicated that *C. rupestre* clustered with other *Chrysanthemum* species, including another former *Ajania* species, *Chrysanthemum pacificum* Nakai, 1928. However, *Ajania variifolia* (C.C.Chang) Tzvelev, 1961, which is a synonym of *Phaeostigma variifolium* (C.C.Chang) Muldashev, 1981, was placed outside the *Chrysanthemum* clade, thereby implying that the former genus *Ajania* includes heterogeneous species.

The genus *Chrysanthemum*, belonging to the tribe Anthemideae within the family Asteraceae, includes cultivated chrysanthemum (*Chrysanthemum morifolium* Ramat., 1792), which is among the most important ornamental flowers (Bremer and Humphries 1993; Ohashi and Yonekura 2004). *Chrysanthemum* species have been classified into four groups, the Indicum, Makinoi, Zawadskii, and Ajania groups, based on their morphological characteristics (Tanaka and Shimotomai 1978). Species in the Ajania group were formerly classified into the genus *Ajania*, which is characterized by a disciform capitulum. However, molecular phylogenetic analyses have revealed that at least some of the species within the former *Ajania* genus form a cluster with the *Chrysanthemum* genus (Masuda et al. 2009; Liu et al. 2012; Nakano et al. 2019). To investigate the relationship between the genera *Chrysanthemum* and the former *Ajania*, we determined the sequence of the whole chloroplast genome of *Chrysanthemum rupestre*, a diploid species belonging to the Ajania group endemic to Japan.

The *C. rupestre* plant (AME15) used for sequencing was collected at Tomi, Japan (N36°21′32.4′′, E138°19′51.5994′′) under permission from Ministry of the Environment of Japan and Tomi city, and is available from National Bioresource Project Chrysanthemum (https://shigen.nig.ac.jp/chrysanthemum/). A voucher specimen (SH1001) and extracted DNA have been deposited in the Herbarium of the Laboratory of Plant Chromosome and Gene Stock, Hiroshima University (contact M. Kusaba, akusaba@hiroshima-u.ac.jp). Total genomic DNA was extracted from fresh leaves of *C. rupestre* using a modified CTAB procedure (Doyle and Doyle 1987). The sequence of the whole chloroplast genome was determined by assembling whole-genome shotgun sequences obtained by using a Novaseq 6000 (Illumina) sequencer in conjunction with GetOrganelle v1.7.4.1 software (Jin et al. 2020). The fastq file extracted using GetOrganelle was submitted to the Genbank SRA database (PRJNA782395). Sequence analysis using the MPI-MP CHLOROBOX GeSeq program (Tillich et al. 2017) revealed that the complete chloroplast genome of *C. rupestre* has a typical conserved quadripartite structure of 151,061 bp in length with an overall GC content of 37.47%, comprising a large single-copy region (LSC, 82,846 bp), a small single-copy region (SSC, 18,301 bp), and a pair of inverted repeat regions (IR, each 24,957 bp), respectively, which are similar to those of another *Chrysanthmum* species, *C. morifolium* cv. Orizaba (LSC, 82,858 bp; SSC, 18,294 bp; IR, each 24,954 bp; and GC content 37.45%; Xia et al. 2021). The chloroplast genome contains 131 genes, comprising 87 protein-coding genes, 8 rRNA genes, and 36 tRNA genes.

Phylogenetic analysis was carried out using whole chloroplast genome sequences of 21 Anthemideae species (Figure 1). Having adjusted the SSC direction, the dataset was aligned using MAFFT v7.490 (Katoh and Standley 2013). The phylogenetic tree was constructed by the maximum likelihood method using PhyML v3.0 with 1000 bootstrap replicates (Guindon et al. 2010). The phylogenetic tree indicated that the genus *Chrysanthemum* forms a single clade with high bootstrap support and a neighboring genus *Artemisia* clade.

**Figure 1.**
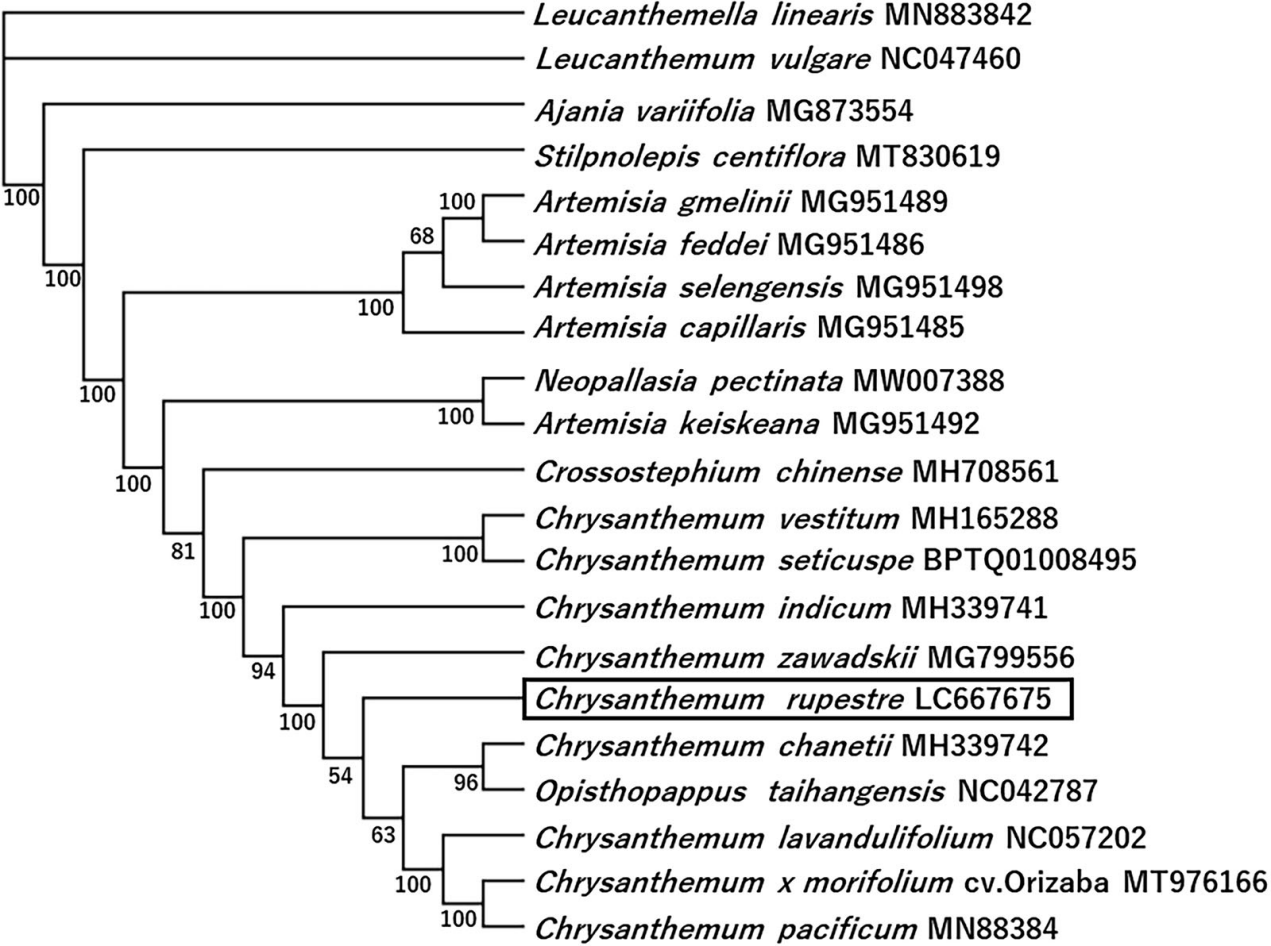
A phylogenetic tree of *Chrysanthemum rupestre* and related species based on complete chloroplast genome sequences. The phylogenetic tree was constructed using maximum-likelihood method with 1000 bootstrap replicates. Names of species and GenBank accession numbers are shown. *Chrysanthemum repestre* is boxed. The bootstrap support values are shown at the branches. Our analysis suggests that *Opisthopappus taihangens*is (Y. Ling) C. Shih,1979, which is synonym to *Chrysanthemum taihangens* Y. Ling, 1939, belongs to the genus *Chrysanthemum*. In our analysis, *Crossostephium chinense* was not clustered with the Artemisia species unlike in the previous report (Chen et al. 2019).

The former *Ajania* species *C. rupestre* and *Chrysanthemum pacificum* are included in the *Chrysanthemum* clade, whereas *Ajania variifolia* (syn. *Phaeostigma variifolium*) is placed outside the *Chrysanthemum* clade. However, Masuda et al. (2009) reported that *P. variifolium* is included in the *Chrysanthemum* clade and distantly related to *Phaeostigma salicifolium* (Mattf.) Muldashev, 1981, implying that the taxonomic position of *P. variifolium* remains to be determined. Nevertheless, our findings indicate that the former genus *Ajania* includes heterogeneous species, comprising at least two distinct groups, namely, *P. variifolium* and *C. rupestre/C. pacificum*.

Furthermore, on the basis of phylogenetic analysis using nuclear-encoded genes, Shen et al. (2021) revealed that *C. pacificum, Chrysanthemum shiwogiku* Kitam., 1935 (syn. *Ajania shiwogiku* (Kitam.) Bremer and Humphries, 1993), and *Chrysanthemum pallasianum* Kom., 1907 (syn. *A. pallasiana* (Fisch. ex Besser) Poljakov, 1955), which are endemic to Japan, fall within the *Chrysanthemum* clade, although other *Ajania* species form a clade separate from the *Chrysanthemum* clade. It was accordingly inferred that the genomes of *C. pacificum*, *C. shiwogiku*, and *C. pallasianum* have been influenced by the introgression of the genome of Indicum complex (Indicum and Makinoi groups) during their evolution. However, molecular phylogenetic analysis based on a nuclear-encoded gene has indicated that *C. rupestre*, a diploid Ajania group species endemic to Japan, forms a clade with *Chrysanthemum potaninii* (Krasch.) Hand.-Mazz., 1938 and *Chrysanthemum nematolobum* Hand.-Mazz.,1938, which are diploid Ajania group species endemic to China (Nakano et al. 2019). Therefore, we anticipate analyses of the whole genome sequences of *C. rupestre* in addition to *Chrysanthemum seticuspe* (Maxim.) Hand.-Mazz., 1936 and *Chrysanthemum makinoi* Matsum. et Nakai, 1916 (Nakano et al. 2021; van Lieshout et al. 2021) may provide a number of clues to clarify the evolutionary history of the *Ajania* group species.

## Data Availability

The genome sequence data that support the findings of this study are openly available in GenBank of NCBI at [https://www.ncbi.nlm.nih.gov] under the accession no. LC667675. The associated BioProject, SRA, and Bio-Sample numbers are PRJNA782395, SRR16996951, and SAMN23377311 respectively.
